# Renal Safety Profile of BCR-ABL Tyrosine Kinase Inhibitors in a Real-Life Setting: A Study Based on Vigibase^®^, the WHO Pharmacovigilance Database

**DOI:** 10.3390/cancers15072041

**Published:** 2023-03-29

**Authors:** Morgane Cellier, Delphine Bourneau-Martin, Chadi Abbara, Alexandre Crosnier, Laurence Lagarce, Anne-Sophie Garnier, Marie Briet

**Affiliations:** 1 Service de Pharmacologie-Toxicologie et Pharmacovigilance, Centre Hospitalo-Universitaire d’Angers, 4 rue Larrey, 49100 Angers, France; 2 Service de Néphrologie, Centre Hospitalo-Universitaire d’Angers, 4 rue Larrey, 49100 Angers, France; 3 UFR Santé, Université d’Angers, 49100 Angers, France; 4 Laboratoire MitoVasc, UMR CNRS 6214 INSERM 1083, 49100 Angers, France

**Keywords:** chronic myeloid leukemia, tyrosine kinase inhibitor, renal adverse effect, nephrotic syndrome, renal artery stenosis

## Abstract

**Simple Summary:**

The development of oral cancer agents known as “tyrosine kinase inhibitors”—imatinib, dasatinib, nilotinib, bosutinib, and ponatinib—has considerably improved patient survival and quality of life in hematological cancers such as chronic myeloid leukemia. These drugs target a fusion protein called BCR-ABL, which is localized in cancer cells. However, these agents also recognize other tyrosine kinases, such as VEGFR, PDFGR, c-KIT, or SRC, for example, that may cause adverse drug effects. Some of these tyrosine kinases are localized in the kidney. These observations raise questions regarding the renal safety profile of BCR-ABL tyrosine kinase inhibitors. This issue was evaluated in the present study, conducted on safety data extracted from VigiBase^®^, the WHO global safety database. This study showed renal failure and fluid retention signals for the five studied drugs and identified new safety signals for nilotinib, with these being nephrotic syndrome and renal artery stenosis.

**Abstract:**

Background: Alongside their BCR-ABL specificity, TKIs used in chronic myeloid leukemia also target other tyrosine kinases expressed in the kidney such as PDGFR, c-KIT, SRC, and VEGFR, which may result in specific renal adverse drug reaction (ADR). To evaluate the renal safety profile in real-life conditions, a case/non-case study was performed on VigiBase^®^, the WHO global safety database. Methods: From 7 November 2001 to 2 June 2021, all cases in which the involvement of imatinib, dasatinib, nilotinib, bosutinib, and ponatinib was suspected in the occurrence of renal ADR were extracted from VigiBase^®^. Disproportionality analyses were assessed using the reporting odds ratio. Results: A total of 1409 cases were included. Imatinib accounts for half of the reported cases. A signal of disproportionate reporting (SDR) of renal failure and fluid retention was found for the five TKIs. Only dasatinib and nilotinib were related to an SDR for nephrotic syndrome. Nilotinib and ponatinib were related to an SDR for renal artery stenosis, while dasatinib was related to an SDR for thrombotic microangiopathy. No SDR for tubulointerstitial nephritis was observed. Conclusion: This study identified a new safety signal, nephrotic syndrome, for nilotinib and highlights the importance of post-marketing safety surveillance.

## 1. Introduction

The development of new anticancer agents, such as targeted therapies, has considerably improved patient survival in hematological cancers such as chronic myeloid leukemia (CML) [1] compared with standard chemotherapy. The molecular pathogenesis of CML is due to a translocation t(9;22), designated as the Philadelphia chromosome (Ph), 22q-, which is detected in 95% of cases of CML and 15 to 30% of cases of acute lymphoblastic leukemia (ALL) in adults [1]. The translocation leads to the BCR-ABL (breakpoint cluster region-Abelson 1) fusion gene that codes for two types of proteins with high tyrosine kinase activity, which is targeted by tyrosine kinase inhibitors (TKIs) [1]. Imatinib was the first TKI targeting BCR-ABL to obtain a marketing authorization (MA) in 2001 for Ph+ CML [2], followed by the development of the second-generation BCR-ABL inhibitors dasatinib, nilotinib, and bosutinib. These three TKIs differ in their potency and activity against BCR-ABL [3,4,5] and were developed to overcome imatinib resistance. Ponatinib, a third-generation TKI, is a therapeutic option for patients with the BCR-ABL^T535I^ mutation and for those with diseases that did not responded to first-line TKIs [6]. CML and ALL represent the main indications of BCR-ABL TKIs (Appendix A). Beside their main target, BCR-ABL TKIs, especially imatinib, also target platelet-derived growth factor receptors (PDGFRs) and c-KIT, which are the basis of other therapeutic indications in myelodysplastic/myeloproliferative diseases with PDGFR re-arrangements, advanced hypereosinophilic syndrome and/or chronic eosinophilic leukemia with FIP1L1-PDGFRα rearrangement, KIT positive malignant gastrointestinal stromal tumors, and dermatofibrosarcoma protuberans care (Table A1) [2,3,4,5,6].

The main reported adverse effects of BCR-ABL TKIs are represented by hematological, gastro-intestinal, neurological, and cardiovascular disorders [2,3,4,5,6,7]. Renal failure has been reported in the literature and in the summary of product characteristics (SmPC) for imatinib, dasatinib, nilotinib, and bosutinib, but without the nature of the kidney damage inflicted being specified, except in the case of dasatinib and its glomerular toxicity [2,3,4,5,6,8,9,10,11,12]. The BCR-ABL TKIs target other tyrosine kinases that are expressed in the kidney [13,14,15] (Table A1). Indeed, imatinib; dasatinib; and, to a lesser extent, nilotinib, bosutinib and ponatinib also inhibit PDGFR, which is expressed in the glomeruli, tubules, renal interstitium, and arteries [13] and is involved in tubular cell reparation, and c-KIT, which has a low level of expression in the collecting duct and distal tubules [13]. Bosutinib and dasatinib inhibit kinases from the SRC family, which are expressed in the renal tubules and, to a lesser extent, in vessels. Ponatinib has an affinity for the vascular endothelial growth factor receptor (VEGFR), which is expressed in the glomeruli, tubules, and vessels [14,15]. VEGFR inhibition has been associated with podocyte damage, resulting in focal segmental glomerulosclerosis and endothelial damage, further resulting in thrombotic microangiopathy. The renal localization of these BCR-ABL TKI off-targets and their role in renal diseases suggest that BCR-ABL TKIs may be responsible for glomerular, tubular, and vascular renal diseases.

Long-term exposure to BCR-ABL TKIs in patients may increase the risk of adverse drug reactions (ADRs) that were not identified in clinical trials, such as renal ADR. In real-life settings, the monitoring of drug safety post-MA is ensured by the pharmacovigilance system, which is based on the principle of spontaneous reporting of ADRs by healthcare professionals or patients [16]. The reports are centralized in national and international databases such as VigiBase^®^, the international pharmacovigilance database of the World Health Organization (WHO).

The aim of this study, conducted on Vigibase^®^ data, was to evaluate the renal safety profile of the five TKIs targeting BCR-ABL—imatinib, dasatinib, nilotinib, bosutinib, and ponatinib—in a real-life setting.

## 2. Materials and Methods

### 2.1. Data Source

This case/non-case study was conducted on data exported from VigiBase^®^, the WHO global database of individual case safety reports (ICSRs). VigiBase^®^ was created by the Uppsala Monitoring Centre (UMC), the WHO’s collaborating center for pharmacovigilance, in 1968 [17]. It is the largest international pharmacovigilance database. Since 1968, more than 20 million reports of suspected ADR have been submitted by member countries of the WHO Programme for International Drug Monitoring [17]. At the time of the data extraction (2 June 2021), 26,463,504 reports of suspected ADRs have been submitted by 159 member countries. The reported data in VigiBase^®^ include pharmacovigilance report characteristics (reporting country and reporter qualification), patient characteristics (gender, age at the time of the effect, medical history, and outcome), the drug information coded according to the Anatomical Therapeutic Chemical (ATC) classification, dosage and time to onset, and ADRs according to the Medical Dictionary for Regulatory Activities (MeDRA^®^) classification. The MeDRA classification is a standardized international medical terminology used in the evaluation of medicinal products. MeDRA terminology is organized into five levels. High-level terms (HLTs) and preferred terms (PTs) are at the third and fourth levels of the MeDRA classification, respectively. PT is a descriptor for a symptom, sign, disease, or diagnosis. PTs can overlap, as is the case, for example, with renal failure and chronic kidney disease. Each PT is linked to at least one HLT [17]. An ICSR was considered serious if one or more of the following criteria were present: hospitalization or prolongation of hospitalization, life-threatening, death, incapacity or disability, congenital abnormality, and other medically significant situations (e.g., change in treatment line resulting in a loss of chance for the patient).

This retrospective study was conducted with the five TKIs targeting BCR-ABL—imatinib (European MA on 7 November 2001), dasatinib (European MA on 20 November 2006), nilotinib (European MA on 19 November 2007), bosutinib (European MA on 27 March 2013), and ponatinib (European MA on 1 July 2013)—from 7 November 2001 to 2 June 2021.

### 2.2. Data Selection

The inclusion criteria for ICSR selection were as follows: ICSRs from 7 November 2001 to 2 June 2021; ICSRs reported by health professionals only; imatinib, dasatinib, nilotinib, bosutinib, or ponatinib as single suspected or interacting drugs; with one of the following “high-level terms” (HLTs) from the broadest MeDRA classification SOC “Renal and Urinary Disorders”: “Glomerulonephritis and nephrotic syndrome”, “Nephritis not elsewhere classified (NEC)”, “Nephropathies and tubular disorders NEC”, “Renal disorders NEC”, “Renal failure and impairment”, “Renal hypertension and related conditions”, and “Renal vascular and ischemic conditions”, and their related preferred terms (PTs). Other HLTs from the SOC Renal and Urinary Disorders were excluded because they were related to urological or infectious diseases. The exclusion criteria were ICSRs reported by a non-healthcare professional responder and ICSRs in which age and sex were not provided according to good pharmacovigilance practice. Duplicates were identified and eliminated by VigiMatch™, a probabilistic record-matching algorithm described and published by Tregunno et al. [18].

### 2.3. Statistical Analysis

After describing the studied population (mean and standard deviation or median and interquartile range for quantitative variables, and count and percent for qualitative variables), a disproportionality analysis was conducted for the following preferred terms (PTs): tubulointerstitial nephritis; acute kidney injury; chronic kidney disease; oliguria; renal failure; renal impairment; fluid retention; renal disorder; nephropathy; toxic nephropathy; nephrotic syndrome; renal artery stenosis; and thrombotic microangiopathy.

For this purpose, the reporting odds ratio (ROR), which is the ratio of the probabilities of the number of ADRs related to one TKI drug compared with those related to other drugs, along with its 95% confidence interval (95% CI), were calculated as follows [19]: *ROR* = (*a*/*c*)/(*b*/*d*) and IC95% = $e^{\log\left(ROR\right)+/-\sqrt{\frac{1}{a}+\frac{1}{b}+\frac{1}{c}+\frac{1}{d}}}$. Here, (a) is the number of ICSRs related to the selected PTs with the suspected TKI, (b) is the number of ICSRs related to the selected PTs with all other drugs, (c) is the number of ADRs other than the selected PTs with the suspected TKI, and (d) is the number of ADRs other than the selected PTs with all other drugs [19]. This enabled the calculation of RORs and 95% CIs by drug and by PT for the studied period. For the disproportionality study, a threshold strictly greater than five cases for a “renal event/TKI” pair was chosen according to the recommendations of the European Medicines Agency (EMA) and the study by Candore et al. [20,21]. A disproportionality signal is identified when the ROR is strictly greater than 1 and the lower bound of its 95% CI is strictly greater than 1 [19].

The software used for analyses was RStudio^®^ version 1.3.1093 for Windows^®^ (version R 4.0.3).

## 3. Results

### 3.1. Characteristics of the 1409 Included Renal Cases

**Figure A1:**
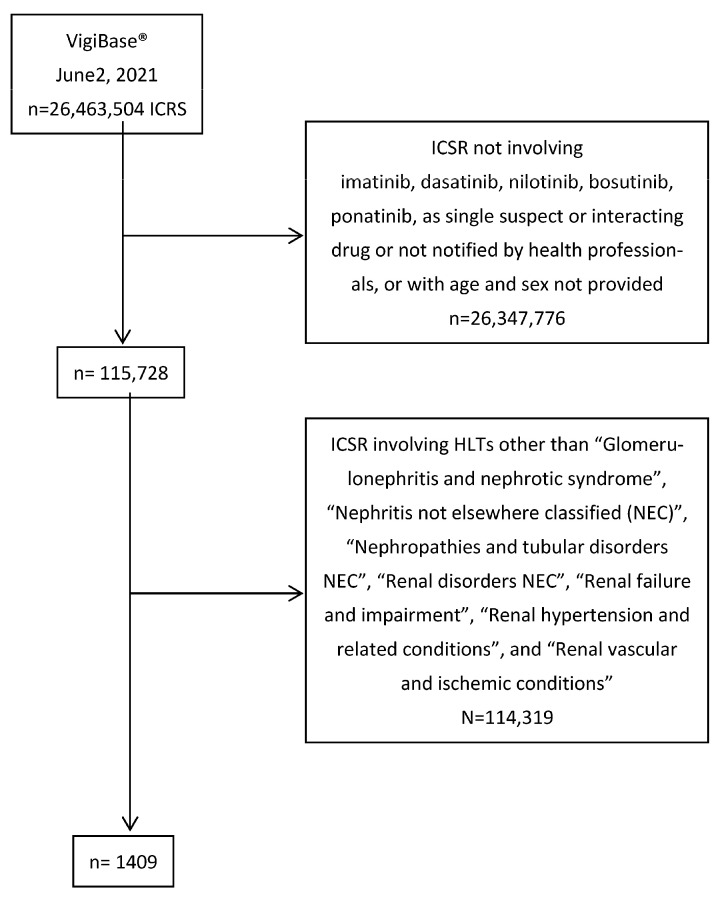
Flow chart of the study.

Of the 26,463,504 ICSRs reported in VigiBase^®^, 115,728 ICSRs referred to imatinib, nilotinib, dasatinib, bosutinib, and ponatinib. Of those, 1409 ICSRs involving 1455 ADRs were related to renal disorders (Figure A1). The main characteristics of the studied cases are presented in Table 1. The majority of the cases involved imatinib (54%), dasatinib (20%), and nilotinib (13%) and were reported by physicians (58%). The mean age was 63.9 (±14.1) years. Fifty-four percent of the cases involved male patients. The majority of the ICSRs were classified as serious (79%, *n* = 1108) and fatalities were reported in 14% of the patients (*n* = 195). The HLTs with the highest number of reported cases were “renal failure and impairment” (*n* = 726, 52%) and “renal disorders” (*n* = 520, 37%), followed by nephropathies and tubular disorders (*n* = 53, 4%), renal vascular and ischemic conditions (*n* = 50, 4%), and glomerulonephritis and nephrotic syndrome (*n* = 39, 3%).

The median times to onset (TTO) for each TKI and ADR are reported in Table 2. The median TTOs varied between 26.5 days and 264.3 days for renal failure and impairment, between 56 days and 383.5 days for renal disorders, between 88 days and 383.5 days for nephropathies and tubular disorders, between 222.8 and 684 days for glomerulonephritis and nephrotic syndrome, and between 61.9 and 616.7 days for renal vascular and ischemic conditions. The ADR was reported as resolved or recovering in 26% of the cases and not resolved or recovered with sequelae in 11% of cases. The evolution was unknown in half of the cases. The suspected TKI was discontinued in 39% of cases (*n* = 557). When reported, the main indication was chronic myeloid leukemia.

### 3.2. Disproportionality Analyses

**Figure 1:**
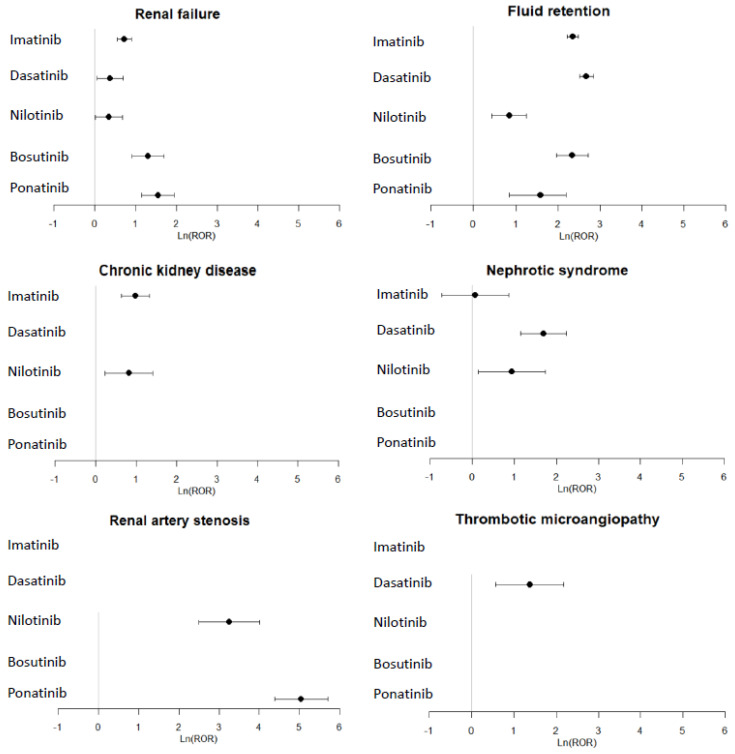
Forest plot of disproportionality (ROR) of tyrosine kinases and renal failure, fluid retention, chronic kidney disease, nephrotic syndrome, renal artery stenosis, and thrombotic microangiopathy events (MeDRA PTs). RORs were calculated when more than five cases were reported for one drug.

The ROR values with the 95% CIs are presented in Figure 1 and in Table A2 for the five BCR-ABL TKIs and the studied PTs.

A significant disproportionality signal was found for renal failure and the five BCR-ABL TKIs. Regarding chronic kidney disease, a signal of disproportionate reporting (SDR) was observed for imatinib (reporting odds ratio (ROR) 2.67; 95% CI: 1.88–3.80) and nilotinib (ROR 2.27; 95% CI: 1.25–4.10). Regarding acute kidney injury, an SDR was displayed only for ponatinib (ROR 1.71; 95% CI: 10.6–2.75). A significant disproportionality signal was observed for fluid retention and the five BCR-ABL TKIs.

Dasatinib and nilotinib were associated with an SDR for nephrotic syndrome (ROR 5.46; 95% CI: 3.16–9.42 and ROR 2.52; 95% CI: 1.16–5.74, respectively).

Regarding renal vascular disease, ponatinib and nilotinib were associated with an SDR for renal artery stenosis (ROR 25.85; 95% CI: 12.20–54.82 and ROR 155.76; 95% CI: 79.94–303.5, respectively), and dasatinib was associated with an SDR for thrombotic microangiopathy (ROR 3.95; 95% CI: 1.77–8.80).

No SDR was displayed for tubulointerstitial nephritis and the five studied BCR-ABL TKIs.

## 4. Discussion

Based on VigiBase^®^ data, this study provides an overview of the renal safety profiles of the TKIs targeting BCR-ABL. Alongside the renal failure and fluid retention signals confirmed for the five studied BCR-ABL TKIs, a nephrotic syndrome signal was identified for dasatinib and nilotinib, a renal artery stenosis signal for nilotinib and ponatinib, and a thrombotic microangiopathy signal for dasatinib.

Concerning the disproportionality study, we preferred to use ROR over methods using the proportional reporting ratio (PRR) and Bayesian methods [19] considering the number of studied cases. A disproportionality analysis is a quick, inexpensive, and sensitive method for screening signals and has its benefits and strengths [22]. The main advantage of a disproportionality analysis is the use of a real-life population, thus limiting patient selection bias. However, this approach needs to be interpreted with caution [22]. A pharmacodynamics hypothesis established upon basic properties of a drug should accompany the interpretation of the data [22].

A fluid retention signal was observed with the five studied TKIs. Fluid retention resulting in anasarca, ascites, and pleural or pericardial effusion is described in the SmPC of the BCR-ABL TKIs and has been extensively reported in the literature [23,24,25,26]. Several mechanisms have been suggested, including the inhibition of PDGFR [26] and alterations of focal adhesions and adherent junctions related to the inhibition of SRC-related kinases [27]. Mahe et al. identified the plausible role of Lyn, an off-target of TKIs, in pleural effusion, through an immune-mediated process [24]. Inhibition of Lyn could destabilize the endothelial barrier, leading to an increased endothelial permeability in pulmonary tissue [24].

**Figure 2:**
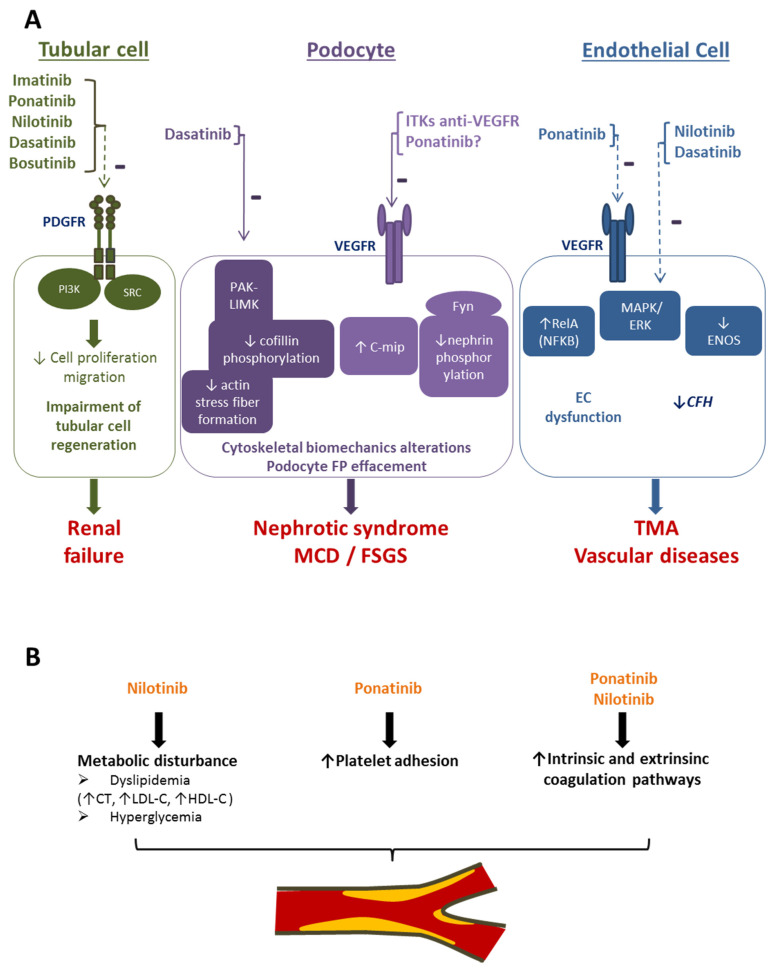
Schematic representation of signaling cascades and their inhibition by BCR-ABL tyrosine kinase inhibitors. (**A**) Renal off-target of BCR-ABL tyrosine kinase inhibitors and the related renal diseases. The five BCR-ABL TKIs exert an inhibitory effect on PDGFR. PDGFR signaling pathways are involved in tubular cell regeneration [29,30]. Dasatinib specifically inhibits PAK–LIMK pathways, resulting in a decrease in cofillin phosphorylation, thus altering actin stress fiber formation and leading to cytoskeletal alterations, podocyte FP effacement, and podocyte loss [12]. Ponatinib may also exert an inhibitory effect on VEGFR [33]. VEGFR inhibition by ITKs targeting this receptor (sunitinib) induces an increase in C-mip and a decrease in nephrin phosphorylation, both leading to cytoskeletal alterations and podocyte disorders, resulting in MCD and FSGS [33,34]. At the level of endothelial cells, ponatinib, dasatinib, and nilotinib interfere directly or indirectly with VEGFR pathways, which may have consequences for EC integrity and function [33]. A decrease in CFH may also be observed, which may favor complement activation [33]. (**B**) Metabolic and coagulation pathway disturbances induced by BCR-ABL tyrosine kinase inhibitors. Nilotinib- and ponatinib-induced metabolic abnormalities [35], platelet adhesion [36], and coagulation pathway activation [37] may contribute to atherosclerotic complications [38]. PDGFR: platelet-derived growth factor; PI3K: phosphatidylinositol 3-kinase; FP: foot process; MCD: minimal change disease; FSGS: focal segmental glomerulosclerosis; C-mip: C-maf-inducing protein; CFH: complement factor H; EC: endothelial cell; TMA: thrombotic microangiopathy (dotted arrows indicate an inhibitory effect reported for the indicated target, but with downstream consequences demonstrated with other TKIs. A solid arrow indicates an inhibitory effect reported for the indicated target with downstream consequences specifically demonstrated for this TKI).

A renal failure signal was also found with the five studied BCR-ABL TKIs. Renal failure is a signal already identified and notified in the SmPC of the five BCR-ABL TKIs. Published cases of renal failure in the context of BCR-ABL TKI exposure showed acute tubular necrosis lesions [28] and could be related to the inhibition of PDGFR. Indeed, the five BCR-ABL TKIs inhibit PDGFR to a greater or lesser extent [2,3,4,5,6]. PDGFR is expressed in the kidney and plays an important role in the proliferation and regeneration of cells in the context of kidney injury [29,30]. Blocking the PDGF/PDGFR pathway may interfere negatively with repair mechanisms and favor the development of renal failure (Figure 2) [31].

Glomerular damage was found with dasatinib and nilotinib, with a “nephrotic syndrome” signal. Nephrotic syndrome is reported in the SmPC of dasatinib [3], but not in the SmPC of nilotinib [4], and is well described in the literature for dasatinib [10,12]. The occurrence of nephrotic syndrome in the context of TKI administration is classically associated with anti-VEGF therapies [15,32]. Minimal change disease and focal segmental glomerulosclerosis are the main diseases resulting in nephrotic syndrome in this context (Figure 2). However, where dasatinib is concerned, the suggested mechanism does not involve VEGFR inhibition, but rather the disruption of actin cytoskeleton and focal adhesion architecture in podocytes owing to LIM kinase pathway inhibition [12]. Calizo et al. showed that dasatinib induces a destabilization of the actin cytoskeleton of podocytes, leading to altered cellular biomechanics through its effect on the PAK–LIMK signaling axis. This effect is not related to VEGFR or SRC inhibition [12] (Figure 2). Regarding nilotinib, the pharmacodynamics explanation of the occurrence of a nephrotic syndrome is less clear because nilotinib targets neither VEGFR nor the LIMK pathways [12]. We hypothesize that the nephrotic syndrome in this case could be a result of the inhibition of the signalization pathway of downstream VEGFR or of hyalinosis developed secondarily to chronic vascular renal lesion. More surprisingly, neither this study nor the literature found any signal of nephrotic syndrome with ponatinib, although this TKI is known to directly inhibit VEGFR [6].

A renal artery stenosis signal was observed with ponatinib and nilotinib. Several studies have indicated that these TKIs are associated with proatherogenic changes, worsening dyslipidaemia (e.g., nilotinib) [35], or a prothrombotic effect due to increasing platelet adhesion (e.g., ponatinib) [36,37,38]. In a murine model, nilotinib and ponatinib were also found to increase the transcription of intrinsic and extrinsic coagulation pathways [37] (Figure 2) and to increase the production of reactive oxygen species from vascular cells [38]. Renal artery stenosis has been well described in the literature and in the SmPC of ponatinib. Regarding nilotinib, cardiovascular events including peripheral artery occlusion disease, ischemic heart disease, basilar artery stenosis, and ischemic cerebrovascular disease have been reported in phase III trials, such as the ENESTnd study (Evaluating Nilotinib Efficacy and Safety in Clinical Trials-Newly Diagnosed Patients), but renal artery stenosis has not been reported yet and is not described in the SmPC [4,38]. However, the rapid evolution of atherosclerosis in this context has led to recommendations regarding ponatinib and nilotinib prescription in patients with pre-existent atherosclerotic risk factors [23]. Altogether, these observations underline the vascular risk associated with ponatinib and nilotinib.

A signal of thrombotic microangiopathy was shown with dasatinib and was already mentioned in its SmPC. This TKI also inhibits the activity of SRC family kinases, which blocks VEGF signaling [15]. This may induce a renal vascular injury phenotype similar to that of other VEGF inhibitors such as sunitinib, sorafenib, pazopanib, and axitinib, for which thrombotic microangiopathy ADR has been well described [15,32].

No renal vascular signal was shown with bosutinib and imatinib. These observations are consistent with the results of numerous trials and post-marketing monitoring showing that imatinib is associated with low cardiovascular toxicity and low arterial thrombotic ADRs, and even with benefits in patients with atherosclerosis [38]. Clinical trials have also shown that bosutinib has similarly low rates of arterial thrombotic ADRs to imatinib [38,39]. The discrepancy between these two TKIs and the other three studied TKIs (dasatinib, nilotinib, and ponatinib) in terms of vascular injury ADR can be explained by their difference in tyrosine kinase specificity [38].

The main limitations of the study arise from the nature of the database, which is based on spontaneous ADR notifications to the pharmacovigilance system. ADRs are usually underreported and the completeness of the information varies between cases and countries [40]. The information comes from a variety of sources and the probability that the suspected adverse effect is drug-related is not the same in all cases. In addition, several others factors may affect the reliability of detecting disproportionality signals such as the time on the market (new or old drugs), the tendency to report only severe adverse events, and selective reporting for a given drug [41,42].

## 5. Conclusions

This is the first study to explore the renal safety profile of BCR-ABL TKIs using a disproportionality analysis based on VigiBase^®^ data. A new safety signal was identified for nilotinib involving nephrotic syndrome. In addition, the five BCR-ABL TKIs were associated with the adverse effects of fluid retention and renal failure. The safety signals nephrotic syndrome and thrombotic microangiopathy were observed with dasatinib and a renal artery stenosis signal was identified for ponatinib and nilotinib.

## Figures and Tables

**Table 1:** Main characteristics of the 1409 cases of nephrotoxicity related to BCR-ABL TKIs.

Parameters	Imatinib *n* = 736	Dasatinib *n* = 287	Nilotinib *n* = 177	Bosutinib *n* = 125	Ponatinib *n* = 84	All Drugs *n* = 1409
**Age in years, mean (** **SD)**	65.0 (13.8)	61.1 (14.4)	62.6 (15.0)	68.1 (11.6)	60.1 (13.6)	63.9 (14.1)
**Sex, *n* (%)**						
Women	328 (45)	143 (50)	69 (39)	68 (54)	35 (42)	643 (46)
Men	408 (55)	144 (50)	108 (61)	57 (46)	49 (58)	766 (54)
**ADR related to renal and urinary disorders in HLT, *n* (%)**						
Renal failure and impairment	393 (53)	104 (36)	104 (59)	74 (59)	51 (61)	726 (52)
Renal disorders	268 (36)	152 (53)	41 (23)	47 (38)	12 (14)	520 (37)
Nephropathies and tubular disorders	39 (5)	0 (0)	9 (5)	2 (2)	3 (4)	53 (4)
Renal vascular and ischemic conditions	15 (2)	8 (3)	10 (6)	0 (0)	17 (20)	50 (4)
Glomerulonephritis and nephrotic syndrome	11 (1)	20 (7)	7 (4)	1 (1)	0 (0)	39 (3)
Nephritis	6 (1)	3 (1)	4 (2)	1 (1)	0 (0)	14 (1)
Renal hypertension and related conditions	4 (1)	0 (0)	2 (1)	0 (0)	1 (1)	7 (1)
**Seriousness, *n* (%)**						
Serious	546 (74)	230 (80)	148 (84)	104 (83)	80 (95)	1108 (79)
Not serious	149 (20)	54 (19)	21 (12)	21 (17)	3 (4)	248 (18)
Unknown	41 (6)	3 (1)	8 (5)	0 (0)	1 (1)	53 (4)
**Outcome, *n* (%)**						
Recovered/resolved	118 (16)	35 (12)	28 (16)	32 (26)	15 (17)	228 (16)
Not recovered	82 (11)	23 (8)	22 (12)	9 (7)	10 (11)	146 (10)
Recovering/resolving	74 (10)	30 (10)	18 (10)	17 (14)	5 (6)	144 (10)
Recovered with sequelae	7 (1)	0 (0)	2 (1)	2 (2)	1 (1)	12 (1)
Death	88 (12)	32 (11)	36 (20)	9 (7)	30 (36)	195 (14)
Unknown	367 (50)	167 (58)	71 (40)	56 (45)	23 (27)	684 (49)
Time to onset in days *, mean (SD)	426 (971)	269 (501)	607 (1041)	127 (327)	524 (627)	410 (880)
Discontinuation of the drug *, *n* (%)	298 (39)	108 (37)	65 (36)	56 (45)	30 (34)	557 (39)
**Top 5 countries reporting cases, *n* (%)**						
United States of America	240 (33)	146 (51)	46 (26)	71 (57)	50 (60)	553 (39)
Japan	124 (17)	28 (10)	37 (21)	20 (16)	9 (11)	128 (15)
Germany	63 (9)	28 (10)	19 (11)	2 (2)	4 (5)	116 (8)
United Kingdom of Great Britain and Northern Ireland	35 (5)	10 (4)	10 (6)	5 (4)	1 (1)	61 (4)
Canada	29 (4)	15 (5)	6 (3)	3 (2)	6 (7)	59 (4)
**Top 3 indications, *n* (%)**						
Chronic myeloid leukemia	343 (47)	179 (62)	127 (72)	72 (58)	37 (44)	758 (54)
Gastrointestinal tumor	132 (18)	0 (0)	2 (1)	0 (0)	1 (1)	135 (10)
Acute lymphoid leukemia	10 (1)	17 (6)	4 (2)	2 (2)	13 (15)	46 (3)

TKI: tyrosine kinase inhibitor; SD: standard deviation; HLT: high-level term; * calculated on the basis of the 1455 adverse drug reactions related to renal and urinary disorders of BCR-ABL TKIs.

**Table 2:** Time to onset and outcome for renal ADR (HLT) and the five studied BCR-ABL TKIs.

		Renal Failure and Impairment (HLT)	Renal Disorders (HLT)	Nephritis (HLT)	Nephropathies and Tubular Disorders (HLT)	Glomerulonephritis and Nephrotic Syndrome (HLT)	Renal Vascular and Ischemic Conditions (HLT)
**Imatinib (*n* = 736)**	**TTO in days, median [Q25–Q75]**	55.0 [13.5–330.3], *n* = 151/393 *	62.0 [11.0–227.0], *n* = 61/268 *	UNK, *n* = 1/6 *	88.0 [28.8–696.0], *n* = 11/39 *	684.0 [14.0–325.5], *n* = 3/11 *	61.9 [29.5–531.0], *n* = 6/15 *
	**Outcome after TKI discontinuation, *n* (%)**						
	Recovered/resolved	74 (19)	32 (12)	0 (0)	5 (13)	2 (18)	2 (13)
	Recovering/resolving	37 (9.4)	25 (9.3)	1 (17)	10 (26)	0 (0)	0 (0)
	Not recovered/not resolved	48 (12)	20 (7.5)	0 (0)	7 (18)	2 (18)	1 (6.7)
	Recovered/resolved with sequalae	5 (1.3)	0 (0)	0 (0)	0 (0)	0 (0)	2 (13)
	Death	39 (9.9)	5 (1.9)	0 (0)	2 (5.1)	0 (0)	2 (13)
	UNK	189 (48)	186 (70)	5 (83)	15 (38.7)	7 (63)	8 (53)
**Dasatinib (*n* = 287)**	**TTO in days, mean (SD)**	264.3 (465.2), *n* = 44/104 *	280.5 (644.8), *n* = 23/152 *	UNK, *n* = 1/3 *	*n* = 0/0 *	222.8 (280.6), *n* = 6/20 *	529.0 (452.4), *n* = 5/8 *
	**Outcome after TKI discontinuation, *n* (%)**						
	Recovered/resolved	13 (12)	14 (9.2)	0 (0)	0 (0)	6 (30)	1 (12)
	Recovering/resolving	8 (7.7)	11 (7.2)	2 (67)	0 (0)	6 (30)	2 (25)
	Not recovered/not resolved	12 (12)	6 (3.9)	0 (0)	0 (0)	1 (5)	2 (25)
	Death	9 (8.7)	0 (0)	0 (0)	0 (0)	0 (0)	0 (0)
	UNK	61 (59)	120 (79)	1 (33)	0 (0)	7 (35)	3 (38)
**Nilotinib (*n* = 177)**	**TTO in days, median [Q25–Q75]**	262.0 [30.7–682.5], *n* = 47/104 *	383.5 [141.3–1071.5], *n* = 14/41 *	UNK, *n* = 1/4 *	383.5 [160.5–591.4], *n* = 4/9 *	366.0 [340.2–632.3], *n* = 4/7 *	616.7 [407.7–1805.9], *n* = 4/10 *
	**Outcome after TKI discontinuation, *n* (%)**						
	Recovered/resolved	15 (14)	7 (17)	2 (50)	1 (11)	0 (0)	2 (20)
	Recovering/resolving	10 (9.6)	3 (7.3)	1 (25)	2 (22)	1 (14)	1 (10)
	Not recovered/not resolved	16 (15)	1 (2.4)	0 (0)	1 (11)	2 (29)	2 (20)
	Recovered/resolved with sequalae	1 (0.96)	1 (2.4)	0 (0)	0 (0)	0 (0)	0 (0)
	Death	19 (18)	4 (9)	0 (0)	1 (11)	0 (0)	0 (0)
	UNK	43 (41)	25 (61)	1 (25)	4 (44)	4 (57)	5 (50)
**Bosutinib (*n* = 125)**	**TTO in days, median [Q25–Q75]**	26.5 [8.0–116.0], *n* = 26/74 *	56.0 [35.0–167.5], *n* = 3/47 *	UNK, *n* = 0/1 *	UNK, *n* = 1/2 *	UNK, *n* = 0/1 *	*n* = 0/0 *
	**Outcome after TKI discontinuation, *n* (%)**						
	Recovered/resolved	22 (30)	9 (19)	0 (0)	0 (0)	0 (0)	0 (0)
	Recovering/resolving	11 (15)	4 (8.5)	1 (100)	1 (50)	0 (0)	0 (0)
	Not recovered/not resolved	6 (8.1)	2 (4.3)	0 (0)	0 (0)	1 (100)	0 (0)
	Recovered/resolved with sequalae	2 (2.7)	0 (0)	0 (0)	0 (0)	0 (0)	0 (0)
	Death	2 (2.7)	0 (0)	0 (0)	0 (0)	0 (0)	0 (0)
	UNK	31 (42)	32 (68)	0 (0)	1 (50)	0 (0)	0 (0)
**Ponatinib (*n* = 84)**	**TTO in days, median [Q25–Q75]**	183.0 [97.5–783.0], *n* = 8/61 *	UNK, *n* = 2/12 *	*n* = 0/0 *	UNK, *n* = 0/3 *	*n* = 0/0 *	490.0 [123.5–800.5], *n* = 7/17 *
	**Outcome after TKI discontinuation, *n* (%)**						
	Recovered/resolved	6 (12)	1 (8.3)	0 (0)	1 (33)	0 (0)	7 (41)
	Recovering/resolving	3 (5.9)	2 (17)	0 (0)	0 (0)	0 (0)	0 (0)
	Not recovered/not resolved	5 (9.8)	2 (17)	0 (0)	0 (0)	0 (0)	1 (5.9)
	Recovered/resolved with sequalae	0 (0)	0 (0)	0 (0)	0 (0)	0 (0)	1 (5.9)
	Death	3 (5.9)	0 (0)	0 (0)	0 (0)	0 (0)	2 (12)
	UNK	14 (66)	7 (58)	0 (0)	2 (66)	0 (0)	6 (34.9)

HLT: high-level term; TTO: time to onset; SD: standard deviation; TKI: tyrosine kinase inhibitor; UNK: unknown. Only HLTs with more than three individual case safety reports appear in this table. * The number of cases for which the TTO is reported is indicated as *n* = number of cases with reported TTO/total number of cases.

## Data Availability

The data presented in this study are available upon request from the corresponding author. The request should be accompanied by a research protocol. The data are not publicly available owing to European ethical and legal restrictions.

## Appendix A

**Table A1:** Market authorizations as well as pharmacokinetic and pharmacodynamic characteristics of BCR-ABL tyrosine kinase inhibitors.

	Imatinib	Dasatinib	Nilotinib	Bosutinib	Ponatinib
BCR-ABL tyrosine kinase inhibitors indications in adult patients	Ph+ CML Ph+ ALL MDS/MDP with PDGFR re-arrangements Advanced HES and/or CEL with FIP1L1-PDGFRα rearrangement Kit (CD 117) positive unresectable and/or metastatic and/or high risk of relapse following resection malignant GISTUnresectable, reccurent or metastatic DFSP not eligible for surgery [2]	Newly diagnosed Ph+ CML in chronic phase, chronic, accelerated, or blast phase CML with resistance or intolerance to previous therapy Ph+ ALL and lymphoid blast CML with resistance or intolerance to previous therapy [3]	Newly diagnosed Ph+ CML in chronic phase, chronic and accelerated phase Ph+ CML with resistance or intolerance to prior therapy No efficacy data available in blast crisis [4]	Newly diagnosed Ph+ CML in chronic phase, chronic, accelerated phase, and blast phase Ph+ CML previously treated with one or more tyrosine kinase inhibitor(s) and for whom imatinib, nilotinib, and dasatinib are not considered appropriate treatment options [5]	Chronic, accelerated, or blast phase Ph+ CML resistant or intolerant to dasatinib or nilotinib, and for whom subsequent treatment with imatinib is not clinically appropriate; or who have the T315I mutation Ph+ ALL, resistant to dasatinib; or intolerant to dasatinib, and for whom subsequent treatment with imatinib is not clinically appropriate; or who have the T315I mutation [6]
**Pharmacodynamics**					
BCR-ABL	++++	++++	++++	++++	++++
Src		+++		+++	
VEGFR					++
PDGFR (α et β)	+++	+	++	+	++
Kit	+++	+++	++	++	++
EphA et EphB		+	+	+	
Lyn				+++	
Hck				+++	
c-FMS (CSF1R)				+	
Txk				+	
Axl				+	
Tec				+	
ErbB				+	
Csk				+	
Stk20				+	
Calmoduline				+	
DDR1 and DDR2	+				
CSF-1	+				
FGFR-1/2/3					
Flt3					+
RET					++
**Pharmacokinetics**					
Dose, mg	400	100	400	200	45
C_max_ Mean (CV%), ng/mL	1606 (40)	104.5 (29)	445 (42)	16.9 (67)	54.7 (26)
T_max_ Median [range], h	2.5 [1.5–6]	0.5 [0.25–1.5]	4 [2–6]	6 [3–36.1]	6 [5–8]
AUC Mean, (CV%), µg·h/mL	25.5 (37)	0.314 (42)	11.9 (56)	0.48 (25)	1.27 (28)
T_1/2_ Mean, (CV%), h	15.7 (18)	3.6 (28)	13 (37)	41.2 (8)	24.2 ^b^ (15)
CL/F Mean (CV%), L/h	18 (32)	0.405 (42)	50.5 (73)	5.60 ^a^ (23)	35.4 (35)
V_d_/F Mean (CV%), L	404 (36)	NA	804 (51)	344 ^a^ (29)	1242 (30)

Ph+ CML: Philadelphia positive chronic myeloid leukemia; Ph+ ALL: Philadelphia positive acute lymphoid leukemia; MDS/MPD: myelodysplastic/myeloproliferative diseases; PDGFR: platelet growth factor receptor; HES: hypereosinophilic syndrome; CEL: chronic eosinophilic leukemia; GIST: gastrointestinal stromal tumors; DFSP: dermatofibrosarcoma protuberans; +/++/+++/++++ indicates the affinity of the TKIs for the indicated target (low, moderate, high, very high); C_max_: peak plasma concentration; T_max_: time to reach peak plasma concentration; AUC: area under the plasma concentration-time curve from zero to infinity; T_1/2_: terminal elimination half-life; CL/F: apparent oral clearance; V_d_/F: apparent volume of distribution. ^a^ CL/F (L/h/Kg), V_d_/F (L/Kg), ^b^ value is median [2,3,4,5,6].

**Table A2:** Reporting odds ratio from standard disproportionality analyses for the selected preferred term (PT).

	Imatinib (*n* = 736)		Dasatinib (*n* = 287)		Nilotinib (*n* = 177)		Bosutinib (*n* = 125)		Ponatinib (*n* = 84)	
	*n*	ROR [CI95low; CI95up]	*n*	ROR [CI95low; CI95up]	*n*	ROR [CI95low; CI95up]	*n*	ROR [CI95low; CI95up]	*n*	ROR [CI95low; CI95up]
**Renal failure and impairment (HLT)**										
Acute kidney injury (PT)	109	0.98 [0.81; 1.19]	37	0.78 [0.56; 1.08]	26	0.56 [0.38; 0.82]	16	1.26 [0.77; 2.07]	17	1.71 [1.06; 2.75]
Chronic kidney disease (PT)	31	2.67 [1.88; 3.80]	5	-	11	2.27 [1.25; 4.10] *	4	-	4	-
Oliguria (PT)	6	0.90 [0.40; 1.99]	2	-	1	-	1	-	0	-
Renal failure (PT)	123	2.07 [1.73; 2.47]	37	1.45 [1.05; 2.00]	35	1.41 [1.01; 1.96]	25	3.69 [2.49; 5.47]	25	4.69 [3.16; 6.96]
Renal impairment (PT)	127	2.39 [2.01; 2.85]	22	0.96 [0.63; 1.46]	26	1.17 [0.79; 1.71]	29	4.79 [3.32; 6.92]	5	-
**Renal disorders NEC (HLT)**										
Fluid retention (PT)	235	10.57 [9.28; 12.04]	139	14.51 [12.26; 17.18]	22	2.30 [1.51; 3.49]	27	10.38 [7.10; 15.18]	10	4.84 [2.30; 9.01]
Renal disorder (PT)	32	2.26 [1.59; 3.19]	8	1.31 [0.66; 2.62]	12	2.02 [1.15; 3.57]	18	11.15 [7.00; 17.74]	2	-
**Nephritis NEC (HLT)**										
Tubulointerstitial nephritis (PT)	6	0.52 [0.23; 1.15]	3	-	2	-	0	-	0	-
**Nephropathies and tubular disorders NEC (HLT)**										
Nephropathy (PT)	13	3.36 [1.95; 5.81]	0	-	2	-	2	-	0	-
Toxic nephropathy (PT)	10	1.28 [0.69; 2.38]	0	-	2	-	0	-	1	-
**Glomerulonephritis and nephrotic syndrome (HLT)**										
Nephrotic syndrome (PT)	6	1.07 [0.48; 2.39]	13	5.46 [3.16; 9.42]	6	2.58 [1.16; 5.74] *	1	-	0	-
**Renal vascular and ischemic conditions (HLT)**										
Renal artery stenosis (PT)	1	-	0	-	7	25.85 [12.20; 54.82]	0	-	9	155.76 [79.94; 303.50]
Thrombotic microangiopathy (PT)	4	-	6	3.95 [1.77; 8.80]	1	-	0	-	4	-

ROR: reporting odds ratio; CI95low: lower boundary of 95% confidence interval; CI95upp: upper boundary of 95% confidence interval; HLT: high-level term; PT: preferred term; in grey, the significant signal. ROR was calculated only for molecules with more than five individual case safety reports. * not reported in the summary of product characteristics.
